# Suicide Risk in Obsessive-Compulsive Disorder: A Case Report

**DOI:** 10.7759/cureus.36863

**Published:** 2023-03-29

**Authors:** José Morais, Mariana Roque, Filipa Santos Martins, Susana Fonseca, Ricardo Moreira

**Affiliations:** 1 Department of Psychiatry, Centro Hospitalar Universitário de São João E.P.E., Porto, PRT

**Keywords:** compulsions, obsessive thoughts, depression, obsessive-compulsive disorder, suicide

## Abstract

Obsessive-compulsive disorder (OCD) is a chronic psychiatric disorder characterized by obsessions and compulsions. It affects about 2.5% of people throughout their life and usually emerges in infancy/adolescence or early adulthood.

Despite high levels of suffering and disability, high comorbidity rates, and low treatment response rates, suicidal behavior associated with this disorder was traditionally considered a rare phenomenon. However, recent studies recognize a significant risk of suicidal behavior in obsessive-compulsive patients.

As a result, we describe a clinical case of attempted suicide in an obsessive-compulsive patient and discuss risk factors that have been considered predictive of suicide in OCD.

## Introduction

Obsessive-compulsive disorder (OCD) is a chronic psychiatric illness characterized by obsessions and/or compulsions [1]. It affects about 2.5% of people throughout their life [2] and usually emerges in infancy/adolescence or early adulthood [1].

Obsessions arise in the patient's mind, recurrently and persistently, as thoughts, impulses, or images experienced as intrusive and unwanted but not as voluntary. Its content is unpleasant or at odds with the patient's values, causing anxiety and significant discomfort. As a form of response to anxiety, the patient typically feels compelled to perform repetitive behaviors or stereotyped mental acts to prevent the unwanted situation or reduce the discomfort caused by the obsession.

Many OCD sufferers have dysfunctional beliefs, including an exaggerated sense of responsibility or a tendency to overestimate a threat. A tendency toward perfectionism and intolerance of uncertainty may also be present, as well as the attribution of excessive importance to thoughts, believing that having a forbidden thought is as bad as acting on it [3].

The degree of insight patients has about their symptoms vary, ranging from excellent or fair to the absence of insight [1].

Obsessions and compulsions are time-consuming and can cause a significant social or occupational deficit with disruption of interpersonal relationships and a significant decrease in quality of life. However, it usually takes patients several years from the onset of symptoms until seeking help. Some studies point to an untreated disease duration of 7 to 9 years, one of the longest among psychiatric illnesses [4].

The variables most associated with the risk of "suicidal behavior" are gender, age, marital status, level of education, and the presence of mental illness.

Despite the high levels of suffering and disability, as well as high rates of comorbidity and, often, poor response to treatment, the suicidal behaviors traditionally associated with this pathology were poorly studied. Self-harmful behaviors related to OCD were considered rare, with completed suicide rates of less than 1% [5]. Some studies even pointed to a negative association between suicide and OCD [6].

About 90% of suicides worldwide are associated with mental illness, especially mood disorders such as major depression, which has the highest comorbidity with OCD [7]. It is estimated that the prevalence of major depression in patients with OCD is 40 to 51%, with a correlation with the duration of the disease [2].

## Case presentation

The patient is a 45-year-old Caucasian male, married, with two daughters, working as a car repairman, who completed the sixth year at the age of 12 through regular education and the ninth year of schooling at 32 through an adult-modified curriculum.

The patient had no significant medical-surgical history. The patient was transferred to the Psychiatry Inpatient Unit from the Cardiothoracic Surgery Unit. He had been hospitalized for two months due to a self-inflicted gunshot wound to the left hemithorax (suicide attempt). Cardiothoracic surgery intervention required correction of cardiac lacerations at the level of the left ventricle and left lung.

In a psychiatric interview, the patient denied any history of suicidal behavior and denied any history of drug abuse. He described himself as an anxious person. Around the age of 20, he developed obsessional thoughts about losing his parents, which he described as having a waxing and waning course, but with long periods of remission. More recently, he reported intrusive and egodystonic thoughts about his sexual orientation in the previous two years that emerged after watching a television report on homosexuality. Despite having never felt sexual attraction to people of the same sex, he mentally reviewed his entire affective and sexual life to be assured that he was not homosexual. These thoughts became increasingly persistent and pervasive. He also mentioned discrimination toward homosexuality in his socio-cultural circle.

He also added obsessive thoughts about the possibility that his daughters might have an accident or die. However, he would not talk to anyone about these thoughts out of shame to avoid worrying the family. At the time, he sought psychiatric help reporting depressive symptoms and was medicated with escitalopram 10mg id, which he abandoned, on his initiative, when he felt better. After a few months, there was a resurgence of obsessive ideas and depressive symptoms, with progressive worsening and associated hopelessness. He decided and planned to end his life, as the suffering associated with the thoughts had become unbearable, and, at that time, he saw no hope or solution for his suffering.

Upon admission to the psychiatric ward, the patient was alert and oriented to person, place, and time. His speech was fluent, logical, and coherent, with regular rhythm and volume. Thought was formally coherent and organized without alterations in possession of thought. The content involved thoughts of death, hopelessness, uselessness, and obsessive doubts about his sexual orientation, generating significant anxiety. There were no perceptual alterations. The mood was depressive, congruent with the content of the thought, and the effects were mood congruent. He had insight into his condition, recognizing the existence of psychopathological changes derived from a mental illness that needed treatment.

The patient was medicated with escitalopram 20mg (to which the patient had a good response in the past and was well tolerated) and olanzapine 5mg a day (off-label to treat anxiety and insomnia, that was suspended after 14 days), with a favorable evolution throughout the hospital stay, with remission of depressive symptoms and suicidal ideation, as well as a decrease in obsessive symptoms.

## Discussion

Although the classic perspective points to a low risk of suicidal behavior in patients with OCD due to characteristic factors such as indecision, difficulty in committing to the act, concern with avoiding aggressive impulses, and preventing self-harm, several more recent studies have pointed to a higher risk of suicide compared to the general population [8-14]. Some studies indicate that around 10% of patients with OCD attempt suicide throughout their lives [15,16].

As observed in the presented clinical case, the obsessive dimension emerges as the main contributory factor to suicide and not so much as the compulsive dimension [17]. Also, obsessions can be associated with a strong feeling of shame, which makes asking for help more difficult and contributes to its aggravation. This cycle can lead to a catastrophic level of anxiety in patients, which can trigger suicidal ideation and behavior [18].

Some sociodemographic variables, such as marital status, professional status, religion, educational level, and family history of suicide, are typically associated with suicide risk. However, in several studies, they are not associated with suicidal behavior in patients with OCD, which makes it difficult to assess the risk in these patients [16,17].

The strongest predictor of suicide in OCD is the previous history of suicidal behavior. Other factors that have been pointed out as predictors of suicide attempts in OCD include comorbidity with depressive and anxiety symptoms, and substance use, the severity of which correlates with higher rates of suicide attempts [8,14] and greater disease severity [3,8,17]. In a meta-analysis conducted by Pellegrini, the severity of the obsessions emerges as a risk factor, and the severity of the compulsions plays a protective factor [14]. This apparent paradox is explained by assigning different neuronal circuits to obsessions and compulsions. While obsessions seem more related to the cortico-striatal-ventro-medial ("affective") tract that modulates emotional regulation and impulse control, compulsions would be more related to the orbitofrontal cortex that controls executive function. According to this theory, greater severity of compulsions would imply a deficit in executive function that would be protective of suicide.

The content of obsessions as a risk factor has shown contradictory evidence in several studies. The aggressive, sexual, and religious content (as in the presented case) has been associated with higher suicidal ideation [19]. At the same time, the meta-analysis conducted by Pellegrini points to a protective role [14].

It is also essential to consider that the obsessive thoughts in impulse phobia may resemble suicidal ideation, which merits careful evaluation. In the case of impulse phobia, these thoughts are typically associated with avoidance behaviors.

There are no treatments aimed at reducing suicidal behavior in OCD. The only agents with a proven reduction in suicide are lithium in bipolar affective disorder and clozapine in schizophrenia, so it is imperative to identify predictive factors of suicide and intervene in modifiable risk factors. The treatment of OCD, which includes psychotherapy, namely cognitive behavioral therapy, and psychopharmacological treatment, namely, with serotonin reuptake inhibitor drugs, thus remains essential for preventing suicide in these patients.

## Conclusions

Patients with OCD are at a higher risk of suicide than the general population, so the psychiatric evaluation must include a careful assessment of suicidal ideation, suicidal plans, or personal history of suicide attempts. This active exploration of suicidal ideation is critical because obsessive themes are often associated with high levels of shame and, therefore, secrecy, as is the case of the patient presented here. The suicide risk increases considerably with comorbidities such as depression, often associated with OCD, as in this case, further reinforces the need for a thorough psychiatric evaluation.
